# Platelet-Activating Factor Acetylhydrolase Expression in BRCA1 Mutant Ovarian Cancer as a Protective Factor and Potential Negative Regulator of the Wnt Signaling Pathway

**DOI:** 10.3390/biomedicines9070706

**Published:** 2021-06-22

**Authors:** Yue Liao, Susann Badmann, Till Kaltofen, Doris Mayr, Elisa Schmoeckel, Eileen Deuster, Mareike Mannewitz, Sarah Landgrebe, Thomas Kolben, Anna Hester, Susanne Beyer, Alexander Burges, Sven Mahner, Udo Jeschke, Fabian Trillsch, Bastian Czogalla

**Affiliations:** 1 Department of Obstetrics and Gynecology, University Hospital, Ludwig Maximilians University (LMU) Munich, 81377 Munich, Germany; yue.liao@med.uni-muenchen.de (Y.L.); susann.badmann@med.uni-muenchen.de (S.B.); till.kaltofen@med.uni-muenchen.de (T.K.); eileen.deuster@med.uni-muenchen.de (E.D.); mareike.mannewitz@med.uni-muenchen.de (M.M.); thomas.kolben@med.uni-muenchen.de (T.K.); anna.hester@med.uni-muenchen.de (A.H.); susanne.beyer@med.uni-muenchen.de (S.B.); alexander.burges@med.uni-muenchen.de (A.B.); sven.mahner@med.uni-muenchen.de (S.M.); udo.jeschke@med.uni-muenchen.de (U.J.); fabian.trillsch@med.uni-muenchen.de (F.T.); 2 Department of Breast Surgery, Zhujiang Hospital Affiliated to Southern Medical University, Guangzhou 510515, China; 3 Institute of Pathology, Faculty of Medicine, Ludwig Maximilians University (LMU) Munich, 81377 Munich, Germany; doris.mayr@med.uni-muenchen.de (D.M.); elisa.schmoeckel@med.uni-muenchen.de (E.S.); sarah.landgrebe@med.uni-muenchen.de (S.L.); 4 Department of Obstetrics and Gynecology, University Hospital Augsburg, 86156 Augsburg, Germany

**Keywords:** platelet-activating factor acetylhydrolase (PAF-AH, PLA2G7), BRCA1 mutant ovarian cancer, Wnt signaling, pGSK3β, β-catenin, prognosis

## Abstract

Aberrantly activated Wnt/β-catenin signaling pathway, as well as platelet-activating factor (PAF), contribute to cancer progression and metastasis of many cancer entities. Nonetheless, the role of the degradation enzyme named platelet-activating factor acetylhydrolase (PLA2G7/PAF-AH) in ovarian cancer etiology is still unclear. This study investigated the functional impact of platelet-activating factor acetylhydrolase on BRCA1 mutant ovarian cancer biology and its crosstalk with the Wnt signaling pathway. PAF-AH, pGSK3β, and β-catenin expressions were analyzed in 156 ovarian cancer specimens by immunohistochemistry. PAF-AH expression was investigated in ovarian cancer tissue, serum of BRCA1-mutated patients, and in vitro in four ovarian cancer cell lines. Functional assays were performed after PLA2G7 silencing. The association of PAF-AH and β-catenin was examined by immunocytochemistry. In an established ovarian carcinoma collective, we identified PAF-AH as an independent positive prognostic factor for overall survival (median 59.9 vs. 27.4 months; *p* = 0.016). PAF-AH correlated strongly with the Wnt signaling proteins pGSK3β (Y216; nuclear: cc = 0.494, *p* < 0.001; cytoplasmic: cc = 0.488, *p* < 0.001) and β-catenin (nuclear: cc = 0.267, *p* = 0.001; cytoplasmic: cc = 0.291, *p* < 0.001). In particular, high levels of PAF-AH were found in tumor tissue and in the serum of BRCA1 mutation carriers. By in vitro expression analysis, a relevant gene and protein expression of PLA2G7/PAF-AH was detected exclusively in the BRCA1-negative ovarian cancer cell line UWB1.289 (*p* < 0.05). Functional assays showed enhanced viability, proliferation, and motility of UWB1.289 cells when PLA2G7/PAF-AH was downregulated, which underlines its protective character. Interestingly, by siRNA knockdown of PLA2G7/PAF-AH, the immunocytochemistry staining pattern of β-catenin changed from a predominantly membranous expression to a nuclear one, suggesting a negative regulatory role of PAF-AH on the Wnt/β-catenin pathway. Our data provide evidence that PAF-AH is a positive prognostic factor with functional impact, which seems particularly relevant in BRCA1 mutant ovarian cancer. For the first time, we show that its protective character may be mediated by a negative regulation of the Wnt/β-catenin pathway. Further studies need to specify this effect. Potential use of PAF-AH as a biomarker for predicting the disease risk of BRCA1 mutation carriers and for the prognosis of patients with BRCA1-negative ovarian cancer should be explored.

## 1. Introduction

Ovarian cancer is one of the five leading causes of cancer-related death in females [1]. Because of minor symptoms at the beginning and limited screening methods for early diagnosis of epithelial ovarian cancer (EOC), the relative 5-year survival rate is less than 45% [2]. Among clinically used prognostic markers, such as the disease stage at diagnosis (FIGO), grading, ascites volume, and patients’ age, the volume of residual disease after surgery is the most relevant [3,4,5]. However, widely accepted non-invasive prognostic biomarkers are rare.

In 15–20% of EOC patients, there is a mutation in the tumor suppressor genes breast cancer 1/2 (BRCA1/2), leading to a familial accumulation of ovarian and breast cancer [6,7]. BRCA genes encode essential caretaker proteins for DNA surveillance and damage repair [8]. When those genes are mutated, damaged DNA may not be repaired correctly via homologous recombination and transcriptional regulation, probably leading to cancer [7,9]. Consequently, BRCA1 mutation carriers have a 40–60% risk of developing ovarian cancer in their lifetime, while BRCA2 mutation carriers’ cumulative risk is up to 25% [10]. Therefore, a genetic examination of BRCA mutation status is indicated in case of positive family history, and close medical care as well as prevention strategies are required.

Platelet-activating factor (PAF) plays a crucial role in inflammation, oncogenic transformation, and metastasis of various tumor entities [11,12,13]. PAF is a lipid second messenger secreted into the tumor microenvironment by circulating cells and cancer cells mediating its effect through a specific G-protein-coupled receptor (PTAFR) [11,14]. Several studies report that PAF and its receptor enhance cancer progression and invasiveness of EOC [15,16,17,18]. Consequently, inhibition of PTAFR leads to sensitization to cisplatin chemotherapy and a reduction in tumor growth [19]. On the basis of this evidence, we hypothesize that an increased degradation of PAF may have protective effects. Therefore, we investigated the role of platelet-activating factor acetylhydrolase (PAF-AH), the degradation enzyme of PAF, in ovarian cancer. PAF-AH is a lipoprotein-bound, calcium-independent phospholipase that is involved in various physiological and pathological processes that influence cell signaling and metabolism [20]. Apart from this, two other PAF-AH types are known in mammals, namely, intracellular type I and II. Even though the PAF-AH isoforms show a low sequence homology, they share a function in PAF catabolism [21]. While intracellular PAF-AH I shows antiapoptotic effects and has often been described as a critical driver in cancer pathogenesis [22,23,24,25], for plasma type PAF-AH, both pro- and anti-tumorigenic effects have been reported. On the one hand, high PLA2G7/PAF-AH expression was associated with aggressive disease and poor prognosis in prostate cancer and in triple-negative breast cancer [26,27]. On the other hand, mouse models of Kaposi’s sarcoma and melanoma with PAF-AH overexpression showed reduced tumor growth and more prolonged survival. In situ, the inactivation of PAF by PAF-AH impaired metastasis through inhibition of neoangiogenesis and tumor cell motility [28].

The canonical Wnt/β-catenin signaling is one of the major pathways involved in tumorigenesis, cancer progression, and the development of therapy resistance to platinum-based chemotherapies or even poly ADP ribose polymerase inhibitors [29,30,31]. The cascade regulates many cellular processes, including development, stemness, cell fate decisions, and cell proliferation [32]. Aberrant activation promotes a wide range of human malignancies, including EOC [33,34,35]. Although mutations in Wnt-related genes are relatively rare in EOC, except for the endometrioid subtype, expression profiling data prove constitutive activation of Wnt signaling in ovarian cancer, most likely by alterations in the subcellular localization of β-catenin [36,37,38]. β-catenin plays a central role in Wnt signaling through its nuclear translocation and activation of β-catenin-responsive genes. It is tightly regulated by its degradation and nuclear translocation [8]. In this context, glycogen synthase kinase-3β (GSK3β) represents an important molecular hub. In its active form (phosphorylated at Y216), GSK3β phosphorylates β-catenin, leading to its ubiquitination and proteasomal degradation [8,39]. Conversely, in the presence of canonical Wnt ligands, GSK3β kinase activity is inhibited by phosphorylation at S9 and nuclear β-catenin levels increase initiating epithelial–mesenchymal transition (EMT) programs [40].

Taken together, the biological consequences of signaling events mediated by PLA2G7/PAF-AH seem to be tissue- and context-dependent and need to be specified in EOC [41]. Beyond the cellular function, we aimed to assess a possible influence of PAF-AH on the Wnt signaling pathway for a better understanding of ovarian cancer pathophysiology, taking into account differences between BRCA1 mutation carriers and BRCA wildtype (WT) patients.

## 2. Materials and Methods

### 2.1. Ethical Approval

The tissue samples used in this study were initially obtained for pathological diagnosis, completed prior to the current study. Patients’ data were fully anonymized and encoded for observers during the analysis procedure. The study was approved by the Ethics Committee of Ludwig Maximilians University, Munich, Germany (approval numbers 227-09, 17-471, 17-527, and 19-972). All experiments were carried out with respect to the standards of the Declaration of Helsinki (1975).

### 2.2. Patients and Specimens

In this study, 156 tissue samples from patients who underwent EOC surgery at the Department of Obstetrics and Gynecology, Ludwig-Maximilian University of Munich, from 1990 to 2002 were analyzed. Patients with benign or borderline tumors were excluded, and no patient had been treated with neoadjuvant chemotherapy. The follow-up data were obtained from the Munich Cancer Registry (Munich Tumor Center, Munich, Germany). The tissue specimens were fixed in 4% buffered formalin and embedded in paraffin for immunohistochemical analysis. Staging and grading of EOC were assessed by gynecological pathologists. Detailed information about the clinical characteristics of patients enrolled in this study, including tumor grading, histology, and staging, was available. The staging was performed according to the WHO and FIGO classification (2014).

Unfortunately, the BRCA mutation status of this EOC collective is not available. Therefore, the BRCA mutation status was defined as unknown, with a BRCA mutation probability of 10–20% [6,7]. To investigate PAF-AH expression levels in BRCA1 mutation carriers, we stained additional tumor tissue of 107 patients with a genetically confirmed BRCA1 mutation (Table 1): 15 patients with a single BRCA1 mutation, and 92 patients with a combined BRCA1/2 mutation.

All mutation carriers showed a proven pathogenic variant and no variant of uncertain significance according to the classification recommended by the IARC Unclassified Genetic Variants Working Group (IARC) and endorsed by the Evidence-based Network for the Interpretation of Germline Mutant Alleles (ENIGMA) Consortium. The specific mutations were identified and evaluated by next-generation sequencing in our genetic laboratory.

Furthermore, blood samples of EOC patients with pathogenic BRCA1 mutation or BRCA WT were used in this study. BRCA mutations were identified by next-generation sequencing in our genetic laboratory. The characteristics of the patients included in blood analysis are shown in Table 2.

### 2.3. Immunohistochemistry and Immunocytochemistry

As previously described, tissue microarrays of formalin-fixed, paraffin-embedded tissue specimens (three spots/patient) were prepared [42]. For immunohistochemistry (IHC) staining, the tissue slides were dewaxed in xylol, washed in 100% ethanol, incubated in methanol with 3% H_2_O_2_ for 20 min, and rehydrated in a descending ethanol gradient. The samples were demasked in a pressure cooker using sodium citrate buffer (pH = 6.0) containing 0.1 M citric acid and 0.1 M sodium citrate in distilled water. After cooking for 5 min, the slides were cooled down and washed in phosphate-buffered saline (PBS). All slides were incubated with a blocking solution for 30 min to prevent the non-specific binding of the primary antibody (Reagent 1; Zytochem-Plus HRP-Polymer-Kit (mouse/rabbit); Zytomed, Berlin, Germany). Primary antibodies against PAF-AH, pGSK3β, and β-catenin (Table S1) were applied for 16 h at 4 °C. The slides were washed with PBS and incubated with a complex of the secondary antibody and an HRP polymer (Reagent 3; Zytochem-Plus HRP Polymer-kit (mouse/rabbit); Zytomed, Berlin, Germany). In order to visualize the immunostaining, we applied the substrate and chromogen-3,3′-diaminobenzidine (DAB; Dako, Hamburg, Germany) for 10 min. The slides were counterstained with Mayer’s hemalum and dehydrated in an ascending series of alcohol. Healthy colon tissue or metastatic colon carcinoma tissue served as positive and negative controls (Figure S1) for the IHC staining to test antibody function and choose the adequate dilution of the antibody.

For immunocytochemistry (ICC), 5 × 103 UWB1.289 cells/cm^2^ were seeded on chamber slides (Merck, Darmstadt, Germany). PLA2G7 silencing of UWB1.289 cells was performed after 48 h incubation. Untreated cells served as reference (basal expression). After treatment, slides were washed with PBS 0.1 M, fixed in 100% ethanol and methanol (1:1) for 15 min at room temperature (RT), and air dried. To reduce non-specific background staining, we treated slides with a protein block solution (Dako, Glostrup, Denmark) for 20 min at RT. The slides were incubated with primary antibodies against PAF-AH and β-catenin (Table S1) for 16 h at 4 °C. After washing with PBS, the slides were incubated with a biotinylated secondary anti-mouse or anti-rabbit antibody (Vector Laboratories, Burlingame, CA, USA) for 30 min at RT. Again, the slides were washed in PBS and incubated with an avidin–biotin peroxidase complex (Vectastain-Elite; Vector Laboratories, Burlingame, CA, USA) for 30 min at RT. The antigen–antibody complex was visualized with the chromogen 3-amino-9-ethylcarbazole (AEC; Dako, Hamburg, Germany) and counterstained with Mayer’s hemalum. Finally, the slides were washed with water and cover slipped using Kaiser’s glycerin gelatin (Merck, Darmstadt, Germany).

### 2.4. Staining Evaluation and Statistical Analysis

For evaluation of PAF-AH, pGSK3β, and β-catenin staining, the semi-quantitative immunoreactive score (IRScore) was used [43], which is calculated by multiplying the optical staining intensity (0 = no, 1 = weak, 2 = moderate, and 3 = strong staining) by the percentage of positive stained cells (0 = no staining, 1 ≤ 10%, 2 = 11–50%, 3 = 51–80% and 4 ≥ 81% stained cells). All slides were analyzed by two independent observers in a double-blind process using a photomicroscope (Leitz, Wetzlar, Germany). The median of IRScores resulting from the three spots of one patient was calculated and used for further analyses.

Data processing and statistical analysis of patient data, IHC results, and blood analysis were performed with SPSS 25.0 (v26; IBM, Armonk, NY, USA). The Mann–Whitney *U* test was applied to compare IRScores or serum concentrations of PAF-AH between two independent subgroups (no/unknown mutation vs. BRCA1) [44]. Spearman’s analysis was used to calculate bivariate correlations between PAF-AH and the Wnt signaling proteins pGSK3β and β-catenin [45]. Survival times were compared using log-rank testing and visualized in Kaplan–Meier plots [46]. To identify appropriate cut-off values ROC analysis, we performed a widely accepted method for cut-off point selection. The Youdan index, defined as the maximum (sensitivity + specificity^−1^) [47], is determined to ensure the optimal cut-off, which maximizes the sum of sensitivity and specificity [48,49]. A Cox regression model was established for multivariate analysis [50]. *p*-values ≤ 0.05 were considered significant. Ct values of the investigated genes were obtained by qPCR and the relative expression was calculated applying the 2-ΔΔCt formula [51]. For data visualization and statistical analysis of in vitro-generated data, Graph Pad Prism 7.03 (v7; San Diego, CA, USA) was used.

### 2.5. PAF-AH ELISA

To determine the PAF-AH concentration in serum samples, we conducted an enzyme-linked immunosorbent assay (ELISA; R&D Systems, Minneapolis, MN, USA) according to the instructions of the manufacturer. The standard curve was created using a four-parameter logistic curve fit. The assay range was 0.8–50 ng/mL with a sensitivity of 0.284 ng/mL.

### 2.6. Cell Lines

The human ovarian cancer cell lines ES-2 (clear cell; ATCC, Rockville, MD, USA), SKOV3 (serous, BRCA WT; ATCC, Rockville, MD, USA), TOV112D (endometrioid; ATCC, Rockville, MD, USA), and UWB1.289 (serous, BRCA1-negative; ATCC, Rockville, MD, USA) were maintained in culture with RPMI 1640 GlutaMAX medium (Gibco, Gibco, Paisley, UK) supplemented with 10% fetal bovine serum (FBS; Gibco, Paisley, UK) in a humified incubator at 37 °C under 5% CO_2_. The benign ovarian cell line HOSEpiC (served as the reference; ATCC, Rockville, MD, USA) was maintained in culture in Ovarian Epithelial Cell Medium (OEpiCM) (ScienCell, Carlsbad, CA, USA,) in a humidified incubator at 37 °C under 5% CO_2_. The benign breast cell line MCF10A (served as the reference; ATCC, Rockville, MD, USA) was maintained in a special growth and assay medium in a humidified incubator at 37 °C under 5% CO_2_.

### 2.7. qPCR

Isolation of mRNA was performed according to the manufacturer’s protocol using the RNeasy Mini Kit (Qiagen, Venlo, The Netherlands). A total of 1 µg RNA was converted into cDNA with the MMLV Reverse Transcriptase 1st-Strand cDNA Synthesis Kit (Epicentre, Madison, WI, USA). qPCR was performed using FastStart Essential DNA Probes Master and gene-specific primers (Roche, Basel, Switzerland). Relative expression was calculated by the 2−ΔΔCt method using β-actin and GAPDH as housekeeping genes (primer sequences are available in the Table S2) [51].

### 2.8. siRNA Knockdown

Lipofectamine RNAiMAX reagent (Invitrogen, Carlsbad, CA, USA) was used to transfect small interfering RNA (siRNA; 4 different sequences for PLA2G7: siRNA 1 (SI00072198): CACCCTTTGGATCCCAAATAA, siRNA 2 (SI00072191): TCAGGACACTTTATTCTGCTA, siRNA 3 (SI00072184): TCCGTTGGTTGTACAGACTTA, siRNA 4 (SI00072177): AAGGACTCTATTGATAGGGAA; Qiagen Sciences, Germantown, MD, USA) into UWB1.289 cells. A scrambled negative control siRNA (Qiagen, Hilden, Germany) was used as a reference. UWB1.289 cells were seeded into 6-well plates, and the transfection was performed when cell density reached 60–70%. The cells were treated with Opti-MEM Reduced Serum Medium (Thermo Fisher Scientific, Waltham, MA, USA) containing siRNA-PLA2G7 and Lipofectamine RNAiMAX. After 36 h, cells were harvested and used for further experiments.

### 2.9. Western Blot

The Western blot analysis was performed as previously reported [52]. In short, adherent cells were lysed for 15 min at 4 °C with 200 µL RIPA buffer (Sigma-Aldrich Co., St. Louis, MO, USA), containing a protease inhibitor (1:100 dilution; Sigma-Aldrich Co., St. Louis, MO, USA). The protein concentration of the lysates was determined with Bradford protein assay. Protein extracts (65 µg) were separated according to their molecular weight using 12% sodium dodecyl sulfate–polyacrylamide gel and transferred onto a polyvinylidene fluoride membrane (EMD Millipore, Billerica, MA, USA). The membrane was blocked for 1 h with casein (Vector Laboratories, Burlingame, CA, USA) to prevent nonspecific binding of the antibodies. After casein saturation, the membrane was incubated with diluted primary antibodies gently shaken overnight at 4 °C. As primary antibodies, a rabbit polyclonal antibody against PAF-AH (1:200 dilution; Cayman, Ann Arbor, MI, USA), a mouse monoclonal antibody against GAPDH (1:1000 dilution; GeneTex Co., Eching, Germany), and a mouse monoclonal antibody against β-actin (1:1000 dilution; Sigma, St. Louis, MO, USA) were used. GAPDH/β-actin Western blots served as controls. Afterwards, membranes were washed with 1:10 casein three times and subjected to biotinylated anti-mouse/anti-rabbit IgG secondary antibodies and ABC-AmP reagent (VECTASTAIN ABC-AmP Kit for rabbit IgG; Vector Laboratories, Burlingame, CA, USA). The antibody complexes were visualized with 5-bromo-4-chloro-3-indolylphosphate/nitroblue tetrazolium chromogenic substrate (Vectastain ABC-AmP Kit; Vector Laboratories, Burlingame, CA, USA). Western blotting detection and analysis was performed with Bio-Rad Universal Hood II and the corresponding software Quantity One (Bio-Rad Laboratories Inc., Hercules, CA, USA). Each Western blot experiment was validated nine times (*n* = 9, three times in three lanes).

### 2.10. Cell Viability Assay and Proliferation Assay

3-(4,5-Dimethylthiazol-2-yl)-2,5-diphenyltetrazolium bromide (MTT) colorimetric assay was performed to measure the cell viability, while 5-bromo-2-deoxyuridine (BrdU) incorporation assay (Roche, Basel, Switzerland) was used to determine cell proliferation. For both assays, 5 × 103 UWB1.289 cells/100 µL were seeded on 96-well plates. The cells were incubated in RPMI 1640 GlutaMAX medium with 10% FBS for 48 h before transfection (PLA2G7 gene knockdown) was performed, as described above. After PLA2G7 36 h gene knockdown, MTT and BrdU assay were conducted according to manufacturer’s protocol. The optical density (OD) was measured with an Elx800 universal Microplate Reader (BioTek, Winooski, VT, USA) at 595 nm (MTT) and 450 nm (BrdU). Each experiment was validated three times (*n* = 3).

### 2.11. Wound Healing Assay

UWB1.289/HCC1937 cells were seeded on a 24-well plate (2 × 105 cells/mL). After 24 h, a vertical line was scratched into the middle of the monolayer with a 100 µL pipet tip to create an artificial wound. Subsequently, the transfection was performed, and digital images of the scratch assays were taken exactly 0 h and 36 h after PLA2G7 gene knockdown. The cell migration was monitored using an inverse phase contrast microscope (Leica Dmi1; Leica, Wetzlar, Germany) with a camera (LEICA MC120 HD; Leica, Wetzlar, Germany). Microphotographs of wounded areas and areas covered with cells were analyzed by ImageJ. Available online: https://imagej.nih.gov/ij/ (accessed on 12 April 2020). The cell migration area is defined as the difference of the area covered with cells at 36 h and 0 h.

## 3. Results

### 3.1. PAF-AH Is an Independent Positive Prognostic Factor in EOC and Correlated with the Wnt Signaling Proteins pGSK3β and β-Catenin

To understand the role of PAF-AH in aberrant cell signaling, we investigated PAF-AH’s expression patterns in 156 EOC specimens by IHC. Similarly, the expression of the Wnt signaling proteins pGSK3β and β-catenin were examined. For PAF-AH, 86.52% of all successfully stained tissue samples were positive with a median (range) IRScore of 3 (0–12). The expression profile of PAF-AH regarding clinical characteristics and pathological data is shown in Table 3. Differences in staining intensity between the histological subtypes were detected. EOC tissue with serous and endometrioid histology showed higher PAF-AH levels than clear cell and mucinous specimens. However, there were no relevant differences regarding subcellular localization of PAF-AH. A total of 98.57% showed cytoplasmatic pGSK3β (Y216; median IRScore = 4 (0–12)), and all specimens showed membranous β-catenin (median IRScore = 8 (2–12)) expression. Strong positive correlations between nuclear/cytoplasmatic PAF-AH, cytoplasmatic pGSK3β, and membranous β-catenin were found (Table 4).

**Figure 1:**
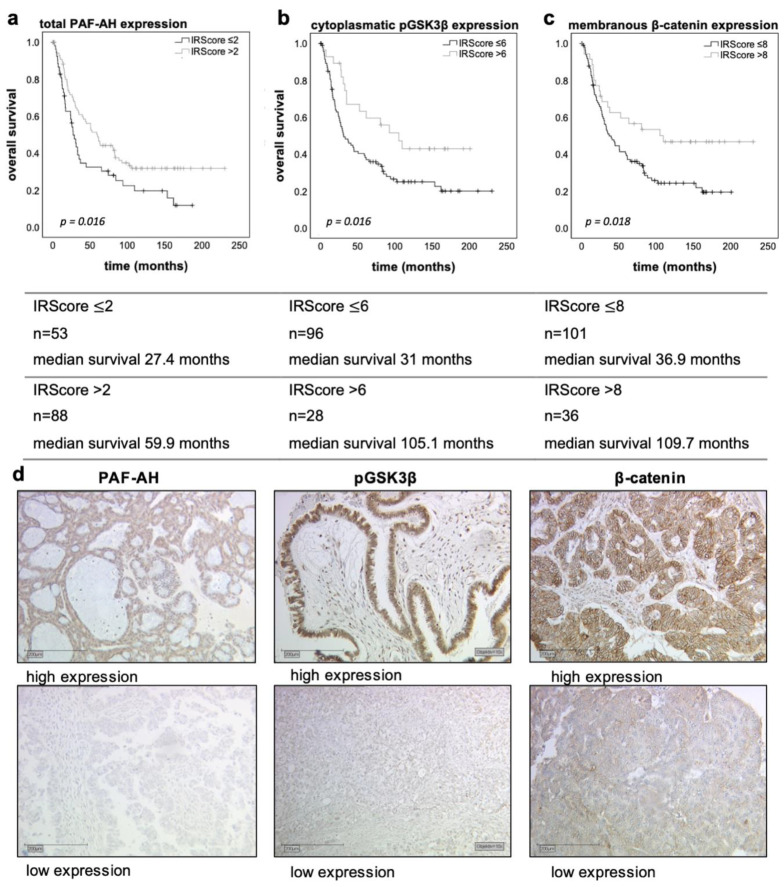
Univariate analyses and representative microphotographs of the immunostainings. The Kaplan–Meier estimates (log-rank testing) show that high tumoral PAF-AH (IRScore > 2; (**a**)) expression as well as high levels of cytoplasmatic pGSK3β (IRScore > 6; (**b**)) and membranous β-catenin (IRScore > 8; (**c**)) are associated with prolonged OS. Censoring events have been marked in the graphs (+). Representative microphotographs of the immunostainings (10× magnification, scale bar = 200 µm; (**d**)) show the difference between high expression (top) and low expression (bottom).

In our patients’ collective, the median age was 58.7 (±31.4) years with a total range of 31–88 years, while the median overall survival (OS) time was 34.4 (±57.8) months. Univariate survival analysis revealed that high levels of all studied proteins are associated with a significantly longer OS (at least twice as long; Figure 1a–c).

Combined survival analysis of the investigated proteins showed an even longer survival time (median OS 199.8 months vs. 35.2 months, *p* = 0.044; Figure S2). However, the subgroup with high expression levels of all factors (total PAF-AH, cytoplasmatic pGSK3β, and membranous β-catenin) was quite small (*n* = 11).

A multivariate Cox regression model was established to assess whether the prognostic factors were independent. Age (>60 vs. ≤60 years, *p* = 0.039), FIGO stage (III/IV vs. I/II, *p* = 0.004), grading (high/G2-3 vs. low/G1, *p* = 0.002), and tumoral PAF-AH expression (high vs. low, *p* = 0.021) turned out to be independent prognostic factors for OS in the present cohort. In contrast, cytoplasmatic pGSK3β (*p* = 0.645) and membranous β-catenin (*p* = 0.745) were not independent (Table 5). Due to insufficient data, the residual disease after primary surgery was not included in the multivariate analysis.

### 3.2. BRCA1 Mutant Patients Had Higher PAF-AH Levels in Tumor Tissue and in Serum

**Figure 2:**
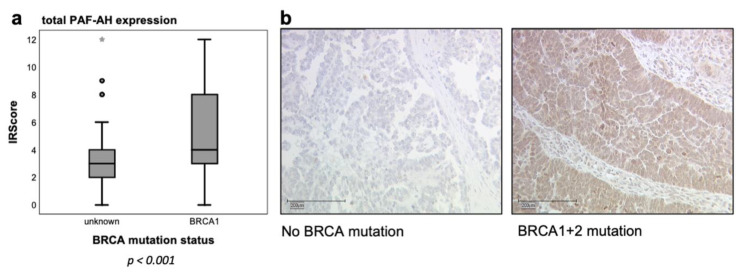
BRCA1 mutation carriers showed higher tumoral PAF-AH expression. PAF-AH expression was significantly higher in tumor tissue of BRCA1 mutation carriers (median IRScore = 4; *p* < 0.001 indicated by asterisk *; (**a**)), when compared (Mann–Whitney *U* test) to patients with unknown BRCA mutation status (median IRScore = 3). Representative microphotographs of PAF-AH immunostaining of patients with BRCA WT and BRCA1 + 2 mutation are shown on the right (**b**) in 10× magnification (scale bar = 200 µm).

PAF-AH expression was also investigated by IHC in tumor tissue of BRCA1 mutation carriers (*n* = 107; Table 2). Interestingly, patients with BRCA1 mutation or BRCA1 + 2 mutations showed significantly higher tumoral expression levels of PAF-AH (median IRScore = 4) compared to patients with unknown BRCA mutation status (*n* = 141; median IRScore = 3), for which a mutation probability of 10–20% can be assumed (Figure 2) [6,7].

**Figure 3:**
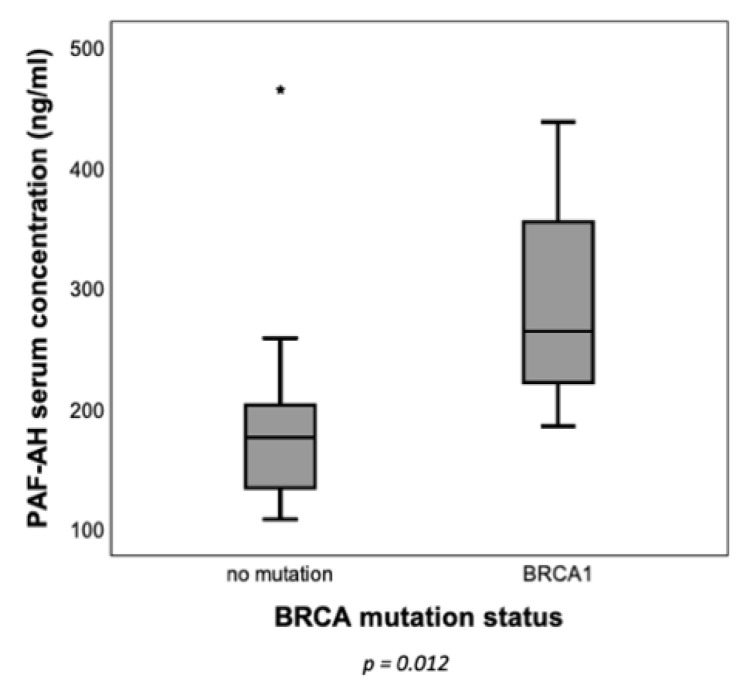
BRCA1 mutation carriers showed higher PAF-AH serum concentrations. PAF-AH serum concentrations were determined with ELISA. Higher levels of PAF-AH were detected in blood samples of BRCA1 mutation carriers (*n* = 6) when compared to patients with BRCA WT (*n* = 17) (Mann–Whitney *U* test, *p* = 0.012 indicated by asterisk *).

On the basis of the results of IHC, the question arose as to whether differences in PAF-AH expression between BRCA WT and BRCA mutation carriers can be detected in blood samples. In a preliminary analysis, PAF-AH serum concentrations of six BRCA1-mutated and 17 BRCA WT EOC patients were determined (Table 2). Indeed, patients with a genetically confirmed BRCA1 mutation had significantly higher PAF-AH serum concentrations (media*n* = 264.56 ng/mL) than BRCA WT patients (media*n* = 176.35 ng/mL, Mann–Whitney *U* test, *p* = 0.012) (Figure 3).

### 3.3. Only BRCA1-Negative UWB1.289 Cell Line Showed Relevant Expression of PLA2G7/PAF-AH

**Figure 4:**
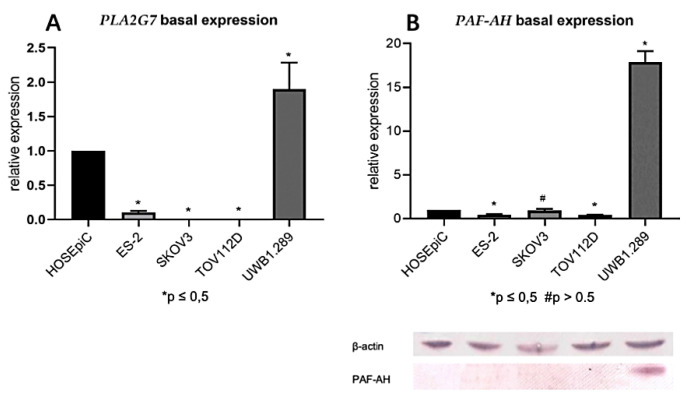
Only the BRCA1 mutant ovarian cancer cell line UWB1.289 showed a relevant expression of PLA2G7/PAF-AH. Basal mRNA (qPCR; (**A**)) and protein (Western blot analysis; (**B**)) expression of PLA2G7/PAF-AH in four ovarian cancer cell lines were compared to the expression in the benign ovarian cell line HOSEpiC. Significant results are indicated by asterisks (*: *p* ≤ 0.05), no significant results by diamonds (#: *p* > 0.05).

The basal mRNA and protein expression of PLA2G7/PAF-AH in four ovarian cancer cell lines were compared to the benign ovarian epithelial cell line HOSEpiC. Both PLA2G7 expression on mRNA level (*p* < 0.05; Figure 4A) and PAF-AH expression on protein level (*p* < 0.05; Figure 4B) were significantly increased in the BRCA1 mutant ovarian cancer cell line UWB1.289 compared to HOSEpiC and other ovarian cancer cell lines. The results derived from qPCR and Western blot were consistent with the results of IHC.

### 3.4. PLA2G7 Knockdown Enhanced Viability, Proliferation, and Motility of UWB1.289 Cells

**Figure 5:**
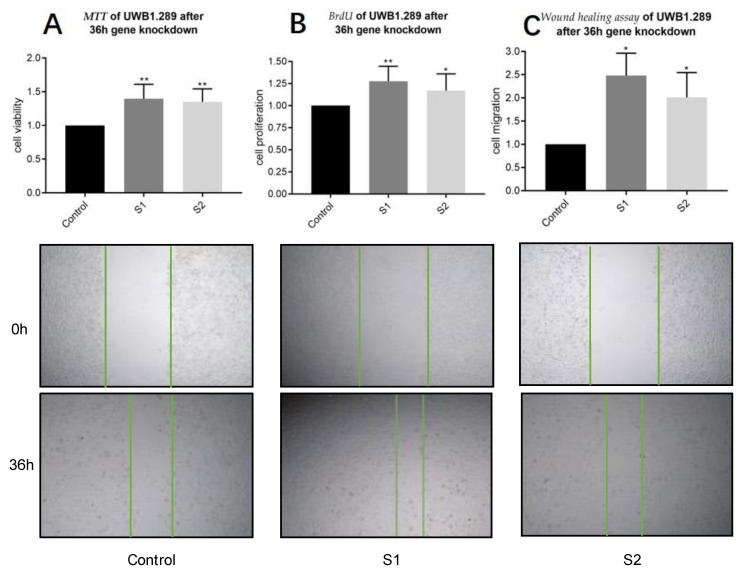
PLA2G7 silencing favored cancer progression by an activation of viability, proliferation, and migration. MTT results show that after 36 h siRNA (sequence S1 and S2) knockdown of PLA2G7, the viability of UWB1.289 increased significantly ((**A**); *p* < 0.001). DNA incorporation of BrdU was also significantly higher in the PLA2G7 downregulated group, indicating an increasing proliferation rate (**B**). The wound healing assay proved that the migration ability of PLA2G7-downregulated UWB1.289 cells was significantly activated compared to the control group ((**C**); *p* < 0.05). Significant results are indicated by asterisks (*: *p* ≤ 0.05) and double asterisks (**: *p* ≤ 0.001).

To assess the functional role of PLA2G7/PAF-AH and its possible impact on the Wnt/β-catenin signaling pathway in ovarian cancer, we performed in vitro experiments in the BRCA1-negative ovarian cancer cell line UWB1.289. Firstly, siRNA was transfected into UWB1.289 for PLA2G7 silencing. A successful downregulation of PLA2G7 and its protein PAF-AH was confirmed by qPCR and Western blot analysis (Figure S3). As a degradation enzyme of PAF, we hypothesized that PAF-AH is a protective factor in ovarian cancer biology. Concordantly, the IHC results showed a positive association of PAF-AH expression with OS. To characterize the cellular function of PAF-AH, we investigated viability, proliferation, and migration of UWB1.289 cells. Results of UWB1.289 cells under PLA2G7 knockdown with the siRNAs described above were compared with the results of an untreated control group (pseudo-knockdown with scrambled siRNA). As shown in Figure 5A, the viability of UWB1.289 cells was increased by PLA2G7 silencing. Furthermore, PLA2G7-downregulated UWB1.289 cells exhibited significantly higher proliferation rates in comparison to the control group, which indicates that PLA2G7 knockdown induces the proliferation of EOC cells (Figure 5B). Results from the wound healing assay showed that after transfection of PLA2G7 siRNA, the migration ability of UWB1.289 was significantly activated compared to the control group (Figure 5C). These results indicate that PLA2G7 silencing causes cancer progression by activation of viability, proliferation, and migration.

### 3.5. The Cellular Distribution Pattern of β-Catenin Changed by PLA2G7 Knockdown from the Membrane to Nucleus

**Figure 6:**
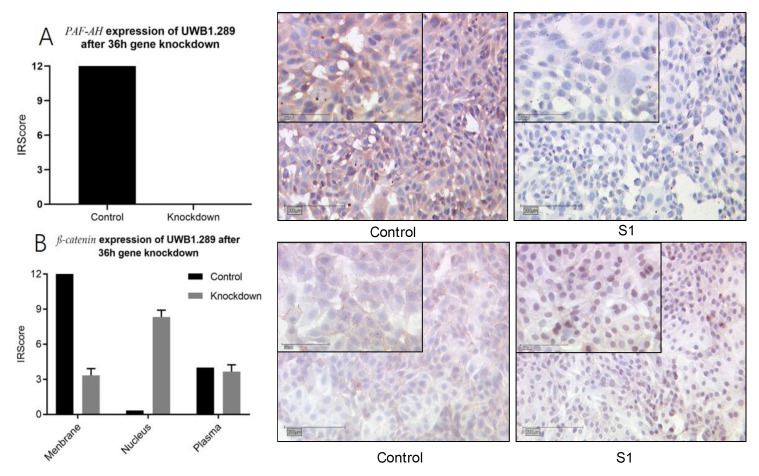
PLA2G7 silencing caused a shift of β-catenin from the membrane to nucleus. ICC staining of PAF-AH and β-catenin changed after 36 h silencing of PLA2G7 with siRNA (representative pictures 1. 10× magnification, scale bar = 200 µm and 25× magnification, scale bar = 100 µm). The expression of PAF-AH was downregulated as expected (**A**). The distribution of β-catenin changed by PLA2G7 knockdown from a predominantly membranous expression to a nuclear one (**B**).

After demonstrating the functional impact of PLA2G7 and its protein PAF-AH on cancer progression, we aimed to validate how PLA2G7 affects the Wnt/β-catenin signaling pathway. On the basis of the correlation of PAF-AH and β-catenin found in IHC, we carried out a series of ICCs to prove an interplay of PAF-AH and β-catenin. As expected, PAF-AH staining was downregulated after knockdown of PLA2G7 compared to the control with pseudo-knockdown (Figure 6A). Interestingly, the distribution of β-catenin also changed by PLA2G7 knockdown. While membrane expression was weakened compared to the control group, the nuclear expression was enhanced (Figure 6B).

## 4. Discussion

In this study, PLA2G7/PAF-AH’s role in ovarian cancer and its influence on the Wnt signaling pathway has been evaluated. Besides cytoplasmatic pGSK3β (Y216) and membranous β-catenin (both part of the inactive state of the Wnt signaling pathway), high tumoral PAF-AH expression was associated with prolonged OS of EOC patients in univariate analysis (Figure 1). A multivariate Cox regression model proved the independence of PAF-AH as a favorable prognostic factor (Table 5). In vitro experiments confirmed protective functional effects of PAF-AH. Silencing of its gene PLA2G7 caused activation of viability, proliferation, and migration of BRCA1 mutant ovarian cancer cells (Figure 5). Since the relevant gene and protein expression of PLA2G7/PAF-AH were detected exclusively in the BRCA1 mutant cell line UWB1.289 (Figure 4), PAF-AH can be considered a new biomarker for BRCA1 mutant ovarian cancer, indicating good prognosis. Significantly higher PAF-AH levels were detected in tumor biopsies (Figure 2) and in the serum of BRCA1 mutation carriers compared to BRCA WT patients (Figure 3). An advantage of PAF-AH as a potential biomarker is the possibility of its non-invasive determination in blood samples before surgery. Since the blood analysis conducted in this study is somewhat preliminary, we suggest further investigation of PAF-AH as a biomarker with prediction ability in BRCA mutant ovarian cancer in a large-scale prospective clinical trial.

**Figure 7:**
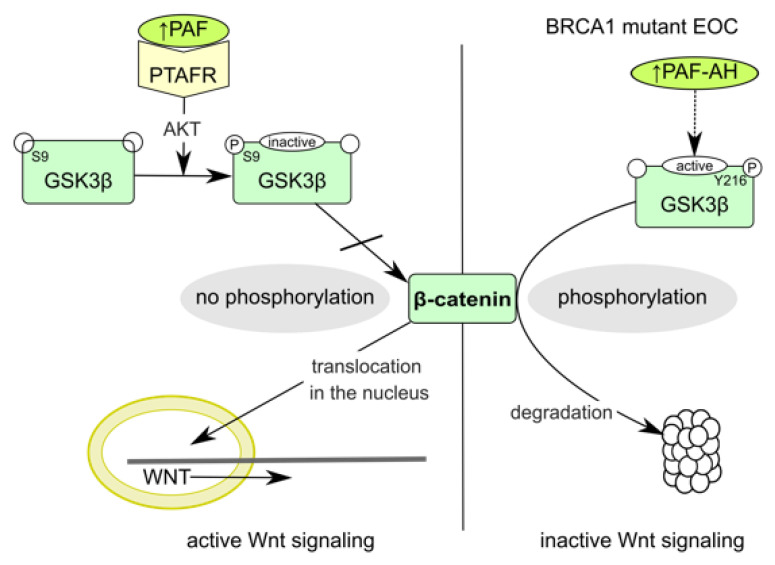
Activation states of canonical Wnt signaling pathway and possible regulation by PAF/PTAFR or PAF-AH. Inactivation of GSK3β by phosphorylation at S9 either in the presence of Wnt ligands or following signal transduction through PAF/PTAFR/AKT (left) led to accumulation of non-phosphorylated β-catenin and translocation in the nucleus [40,53]. There, β-catenin displaces Groucho/TLE repressors from transcription factors Tcf/Lef and activates transcription of Wnt-responsive genes [54]. In the absence of Wnt ligands or PAF signaling, e.g., through enhanced PAF degradation by PAF-AH (right), β-catenin is marked by active GSK3β for ubiquitination and proteasomal degradation [8,39].

We further show that PAF-AH expression positively correlated with cytoplasmatic pGSK3β (Y216) and membranous β-catenin expression, which suggests an interaction with the Wnt/β-catenin signaling pathway. A changed distribution pattern of β-catenin within the cellular departments in BRCA mutant ovarian cancer cells caused by PLA2G7 gene knockdown confirmed this assumption. Membrane expression of β-catenin was reduced, while nuclear expression was upregulated (Figure 6). Thus, increased activation of the Wnt signaling could be responsible for tumor progression under PLA2G7 knockdown. We assume that PAF/PTAFR and PLA2G7/PAF-AH might have a negative regulatory influence on the Wnt signaling pathway, especially in BRCA1 mutant EOC (Figure 7) [53].

Similarly, studies of Furihata et al. [55] and Boccellino and Camussi et al. [56] indicate a functional link between PAF/PTAFR and β-catenin. A PTAFR antagonist reduced inflammation-induced colon carcinogenesis in rats, and β-catenin was localized in the cell membrane in healthy tissue, while it was overexpressed in the nucleus in precursor lesions and colon cancer [55]. Immunofluorescence analysis of Kaposi’s sarcoma cells also showed a change in β-catenin distribution from the membrane to a diffuse pattern as a reaction to PAF treatment [56].

On the basis of our findings and evidence from literature, we can consider two explanatory approaches for the protective effects of PAF-AH: (1) Influence of PAF-AH on the Wnt signaling pathway by regulating PAF levels in the tumor microenvironment. (2) PAF independent regulatory effect of PAF-AH on Wnt downstream genes. Indeed, the anti-inflammatory properties of PAF-AH further contribute to its protective character [57].

**Figure 8:**
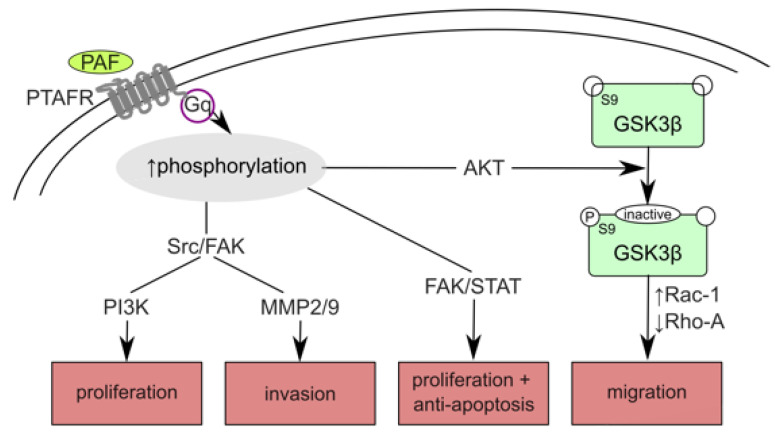
Functional consequences of PAF-AH silencing. By PLA2G7 silencing, PAF-mediated signaling dominated. Binding to its Gq-coupled receptor PTAFR, PAF activated phosphorylation cascades, which often lead to oncogenic transformation, tumor growth, angiogenesis, and metastasis [15,53,60,61]. Activation of Src/FAK and their downstream targets PI3K and MMP2/MMP9 resulted in cancer cell proliferation and cancer invasion, respectively [15]. Proliferation and anti-apoptosis are induced via FAK/STAT [61]. GSK-3β is inactivated by AKT, leading to enhanced migration by Rac-1 activation and Rho-A inactivation and active Wnt signaling by stabilizing β-catenin [53].

PAF induces various signaling pathways via its G-protein-coupled receptor PTAFR through the activation of phosphorylation cascades [15,58]. These phospholipid-mediated protein phosphorylation cascades often represent early responses to mitogenic induction [59]. While PAF exposure activates, e.g., Src/FAK, FAK/STAT, and AKT, leading to enhanced proliferation, invasion, and migration, respectively (Figure 8) [15,53,60,61], GSK3β is inactivated by phosphorylation at S9 [53,60]. β-Catenin is thereby stabilized and activates Wnt-responsive genes (Figure 7).

Zhang et al. reported an elevated expression of PTAFR in BRCA1 mutant cell lines and tissue of BRCA1 mutation carriers. Additionally, they showed PAF/PTAFR-mediated malignant transition of BRCA1-mutated non-malignant ovarian epithelial cells by FAK/STAT phosphorylation, thereby inducing proliferation and anti-apoptosis [61]. As we also found higher PAF-AH levels in BRCA1 mutation carriers and BRCA1 mutant ovarian cancer cells, we conclude that PAF-AH upregulation might be relevant to counteract PAF and Wnt signaling, respectively. By increased PAF degradation, GSK3β remains active, and β-catenin is marked for degradation, resulting in an inactive Wnt pathway (Figure 7) [39]. The same effect was observed for PTAFR antagonism [61].

Direct modulation of the Wnt signaling pathway by the catalytic subunits of intracellular PAF-AH isoform IB was discovered by Livnat et al. in restricted areas of the cerebral cortex [62]. In addition to PAF degradation, it is conceivable that the PAF-AH isoforms show other parallels on a regulatory level. In line with our results, Livnat et al. showed enhanced proliferation and tangential migration of GABAergic interneurons in PAF-AH knockout mice. Overexpression of each of the catalytic subunits provoked a shift of β-catenin from the nucleus to the cytoplasm and repressed Wnt gene expression [62]. However, the molecular interaction between PAF-AH and β-catenin remains unclear and needs to be defined in future studies.

For breast cancer, an interplay between BRCA1 and the Wnt signaling pathway has been previously described. Wu et al. found an inverse correlative association between Wnt signaling and BRCA1 expression in basal-like breast cancer due to epigenetic repression of BRCA1 by the Wnt effector Slug [40]. Li et al. reported that the nuclear form of β-catenin was lower or absent in most BRCA1 familial breast cancer tissues compared to sporadic breast cancer or healthy tissue [8]. For BRCA1 WT, but not mutated BRCA1, direct interaction with β-catenin on the same binding site as the ubiquitinylating enzyme was described. Consequently, the half-life of β-catenin is prolonged, and the Wnt signaling pathway is active in the presence of BRCA1 WT [8]. In BRCA mutant ovarian cancer, Wnt signaling might be repressed by PAF-AH. Nevertheless, we cannot exclude participations or crosstalk with other signaling pathways and regulatory factors resulting in the observed phenotypes.

## 5. Conclusions

Patients and in vitro generated data indicate that PAF/PTAFR and PLA2G7/PAF-AH signaling plays a crucial role in BRCA1 mutant ovarian cancer. While PAF and its receptor PTAFR promote malignant transformation [59,61], tumor progression [12,15,55,63], and chemoresistance [19], we were able to identify PLA2G7/PAF-AH as counterpart and protective factor. Strong positive correlations between PAF-AH and the Wnt signaling proteins pGSK3β and β-catenin and a shift of β-catenin from the membrane to the nucleus by PLA2G7 silencing suggest a negative regulatory impact of PAF-AH on the Wnt/β-catenin signaling pathway. Since PAF-AH mediates protective effects and is non-invasively detectable in blood samples, it should be considered a potential biomarker that indicates a good prognosis for patients with BRCA1 mutant ovarian cancer. Thus, further studies are needed to validate our findings.

## Figures and Tables

**Table 1:** BRCA mutation status of analyzed patients.

BRCA Mutation Status	*n*	Percentage (%)
Mutation unknown	141	56.9
BRCA1 mutation	107	43.1
BRCA1	15	6
BRCA1 + 2	92	37

**Table 2:** Patient characteristics of the blood analysis.

BRCA Mutation Status	*n*	Percentage (%)	Overall Survival (Months; Median)	Progression-Free Survival (Months; Median)
No mutation	17	73.9	25.0	17.0
BRCA1 mutation	6	26.1	34.5	28.0

**Table 3:** Expression profile of PAF-AH regarding clinical and pathological characteristics.

Clinicopathological Parameters	PAF-AH Total			PAF-AH Nucleus			PAF-AH Cytoplasm		
	*n*	Median IRScore	*p*	*n*	Median IRScore	*p*	*n*	Median IRScore	*p*
**Histology**			<0.001 *			<0.001 *			<0.001 *
Serous	102	3		102	3		102	3	
Clear cell	11	1		11	1		11	1	
Endometrioid	19	3		19	2		19	3	
Mucinous	10	0.5		10	0.5		10	1	
**Lymph node**			NS			NS			NS
pN0/X	94	3		94	2		94	3	
pN1	48	3		48	2		48	3	
**Distant Metastasis**			NS			NS			NS
pM0/X	137	3		137	2		137	3	
pM1	5	4		5	2		5	3	
**FIGO**			NS			NS			NS
I/II	39	3		39	2		39	3	
III/IV	98	3		98	2		98	3	
**Age**			0.047 *			0.022 *			0.047 *
≤60 years	75	3		75	2		75	3	
>60 years	67	3		67	3		67	3	
**Serous Grading**			NS			NS			NS
Low	22	3		22	3		22	3	
High	74	3.5		74	3		74	3	
**Clear cell, endometrioid, mucinous grading**			NS			NS			NS
G1	10	2.5		10	1		10	2.5	
G2	10	2		10	1		10	2.5	
G3	15	2		15	1		15	2	

Differences in IRScores of PAF-AH (total, nucleus and cytoplasm) staining were detected regarding clinical and pathological characteristics using Mann–Whitney *U* test. Significant results are indicated by asterisks (*: *p* ≤ 0.05). *p* = two-tailed significance, *n* = number of patients, NS = not significant.

**Table 4:** Correlations between PAF-AH and Wnt signaling proteins pGSK3β and β-catenin.

	PAF-AH Nucleus	PAF-AH Cytoplasm	pGSK3β Cytoplasm	β-Catenin Membrane
PAF-AH nucleus				
Cc	1	0.469	0.494	0.267
*p*	-	<0.001 *	<0.001 *	0.001 *
*n*	141	141	135	140
PAF-AH cytoplasm				
	0.469	1	0.448	0.291
	<0.001 *	-	<0.001 *	<0.001 *
	141	141	135	140
pGSK3β cytoplasm				
Cc	0.494	0.448	1	0.224
*p*	<0.001 *	<0.001 *	-	0.008 *
*n*	135	135	140	139
β-Catenin membrane				
Cc	0.267	0.291	0.224	1
*p*	0.001 *	<0.001 *	0.008 *	-
*n*	140	140	139	147

IRScores of PAF-AH (nucleus and cytoplasm), pGSK3β (cytoplasma) and β-catenin (membrane) staining were correlated to each other using Spearman’s correlation analysis. Significant correlations are indicated by asterisks (*: *p* ≤ 0.05). Cc = correlation coefficient, *p* = two-tailed significance, *n* = number of patients.

**Table 5:** Multivariate analysis confirmed the independency of tumoral PAF-AH expression as a positive prognostic factor for OS.

Covariate	*p*	Hazard Ratio (95% CI)
Age > 60 vs. ≤60	0.039 *	1.637 (1.026–2.612)
FIGO III/IV vs. I/II	0.004 *	2.585 (1.366–4.891)
Grading high/G2-3 vs. low/G1	0.002 *	2.797 (1.436–5.449)
Total PAF-AH expression high (>2) vs. low (≤2)	0.021 *	0.583 (0.369–0.921)
Cytoplasmatic pGSK3β (Y216) expression high (>6) vs. low (≤6)	0.645	0.877 (0.501–1.535)
Membranous β-catenin expression high (>8) vs. low (≤8)	0.745	0.736 (0.545–1.544)

Significant independent factors are indicated by asterisks (*: *p* ≤ 0.05). CI: confidence interval.

## Data Availability

The datasets generated and/or analyzed during the current study are available from the corresponding author on reasonable request.

## Supplementary Materials

The following are available online at https://www.mdpi.com/article/10.3390/biomedicines9070706/s1, Table S1: Antibodies used for immunostainings. Table S2: Sequences of primers used in qPCR to determine mRNA expression levels. Figure S1: Positive and negative system controls. Figure S2: Combined survival analysis of PAF-AH, pGSK3β (cytoplasmatic), and β-catenin (membranous). Figure S3: Successful PLA2G7/PAF-AH downregulation by siRNA knockdown.

## Abbreviations

ADP	adenosine diphosphate
AKT	protein kinase B
BRCA	breast cancer gene
BrDU	5-bromo-2-deoxyuridine
Cc	correlation coefficient
CI	confidence interval
DNA	deoxyribonucleic acid
ELISA	enzyme-linked immunosorbent assay
EMT	epithelial–mesenchymal transition
EOC	epithelial ovarian cancer
FBS	fetal bovine serum
FIGO	International Federation of Gynecology and Obstetrics
GAPDH	glycerinaldehyd-3-phosphat-dehydrogenase
GSK3β	glycogen synthase kinase-3β
ICC	immunocytochemistry
IHC	immunohistochemistry
IRScore	immunoreactive score
MMP	matrix metalloproteinase
mRNA	messenger ribonucleic acid
MTT	3-(4,5-dimethylthiazol-2-yl)-2,5-diphenyltetrazolium bromide
OS	overall survival
PAF	platelet-activating factor
PBS	phosphate-buffered saline
PI3K	phosphatidylinositol 3-kinase
PLA2G7/PAF-AH	platelet-activating factor acetylhydrolase
PTAFR	platelet-activating factor receptor
qPCR	quantitative polymerase chain reaction
Rac-1	ras-related C3 botulinum toxin substrate 1
Rho-A	ras homolog family member A
RIPA	radioimmunoprecipitation assay
ROC	receiver operating characteristic
RT	room temperature
siRNA	small interfering ribonucleic acid
Src/FAK	steroid receptor coactivator/focal adhesion kinase
STAT	signal transducer and activator of transcription
Tcf/Lef	transcription factor/lymphoid enhancer-binding factor
TLE	transducin-like enhancer
WHO	World Health Organization
WT	wildtype
