# Supplementary material for: Platelet-Activating Factor Acetylhydrolase Expression in BRCA1 Mutant Ovarian Cancer as a Protective Factor and Potential Negative Regulator of the Wnt Signaling Pathway
Source: Biomedicines. 2021 Jun 22;9(7):706. doi: 10.3390/biomedicines9070706 (PMC8301368; doi:10.3390/biomedicines9070706)
Supplement: Supplementary file 1 [file biomedicines-09-00706-s001.zip › biomedicines-1233939-SI.pdf]

Supplementary Materials

Platelet-activating Factor Acetylhydrolase Expression in BRCA1 Mutant Ovarian Cancer as a Protective Factor and Potential Negative Regulator of the Wnt Signaling Pathway

Table S1: Antibodies used for immunostainings.

Antibody	Dilution	Manufacturer
**anti-PAF-AH, monoclonal mouse**	1:200 (IHC)<br>1:25 (ICC)	MyBioSource, San Diego, CA, USA
**anti-GSK3β (phospho Y216), polyclonal rabbit**	1:1000 (IHC)	Abcam, Cambridge, UK
**anti-β-catenin, polyclonal rabbit**	1:300 (IHC)<br>1:200 (ICC)	Diagnostic BioSystems, Fremont, CA, USA

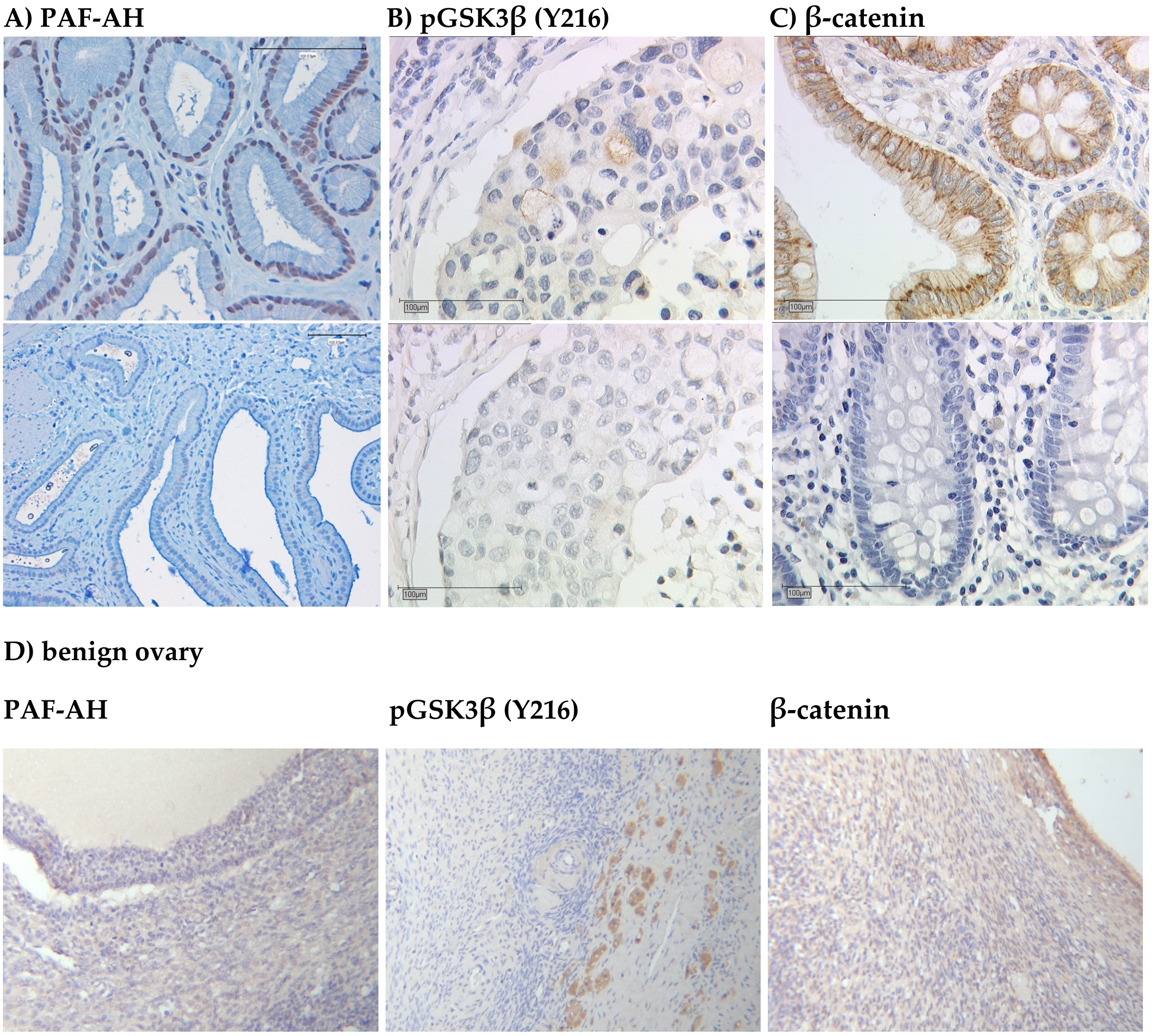

Figure S1: Positive and negative system controls. (**A–C**) Metastatic or healthy colon tissue served as positive (top) and negative (bottom) system controls for immunostainings (25× magnification). (**D**) Staining of benign ovary (10× magnification).

Table S2: Sequences of primers used in qPCR to determine mRNA expression levels.

**PLA2G7**	Forward: GGCTCTACCTTAGAACCCTGAAA
	Reverse: TTTTGCTCTTTGCCGTACCT
**β-actin**	Forward: TCCTCCCTGGAGAAGAGCTA
	Reverse: CGTGGATGCCACAGGACT
**GAPDH**	Forward: AGCCACATCGCTCAGACAC
	Reverse: GCCCAATACGACCAAATCC

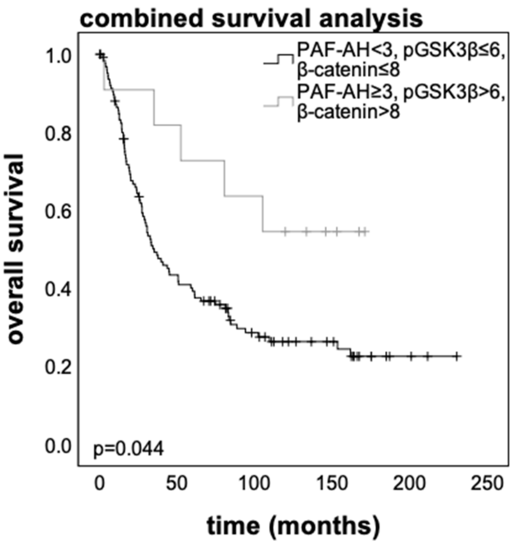

low expression of all proteins<br>n = 128<br>median survival 35.2 months
high expression of all proteins<br>n = 11<br>median survival 199.8 months

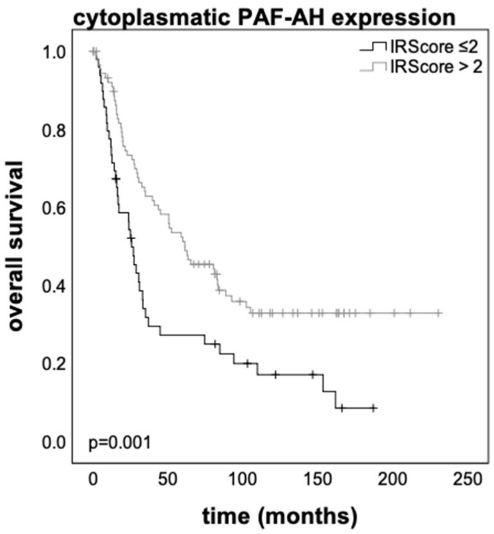

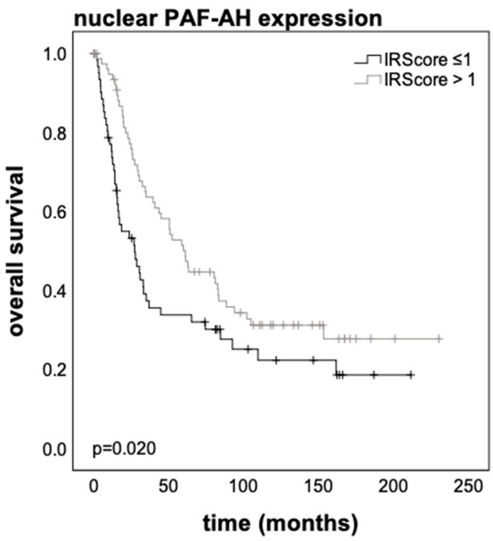

IRScore ≤ 2<br>n = 49<br>median survival 25.7 months	IRScore ≤ 1<br>n = 62<br>median survival 27.4 months
IRScore > 2<br>n = 92<br>median survival 61.3 months	IRScore > 1<br>n = 79<br>median survival 61.2 months

Figure S2: Combined survival analysis of PAF-AH, pGSK3β (cytoplasmatic), and β-catenin (membranous). The Kaplan Meier estimate (log-rank testing) shows that high tumoral expression levels of the investigated proteins are associated with prolonged OS. Censoring events have been marked in the graph (+).

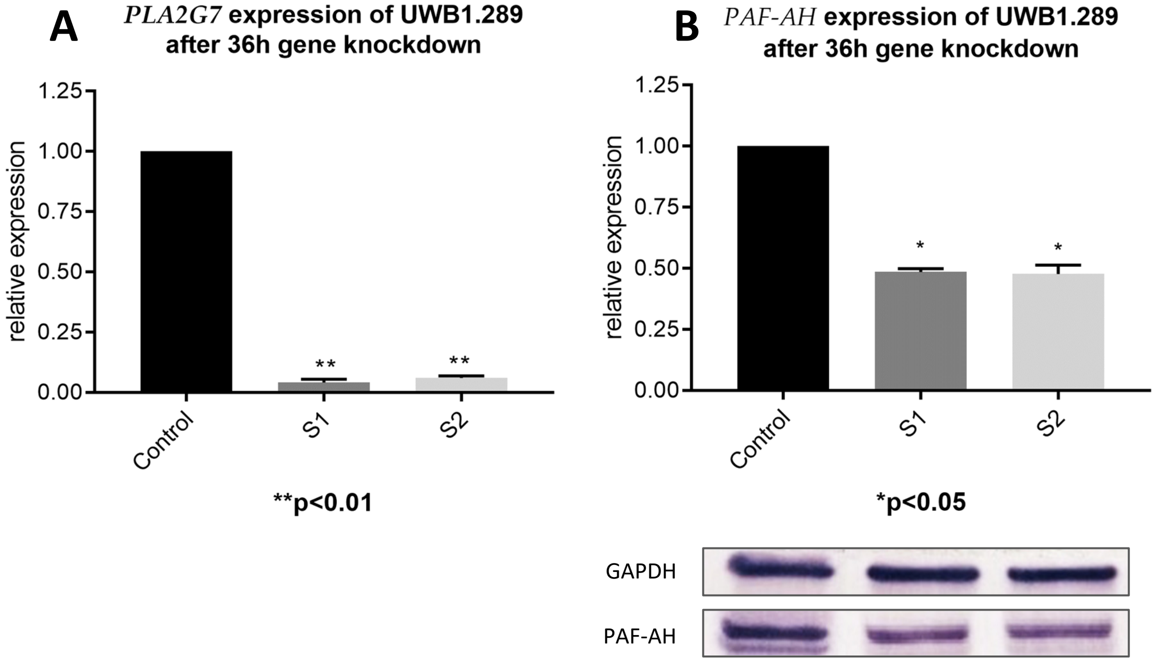

Figure S3: Successful PLA2G7/PAF-AH downregulation by siRNA knockdown. The efficiency of siRNA knockdown was investigated by qPCR (**A**) and western blot analysis (**B**). The best knockdown of PLA2G7 was achieved after an incubation time of 36h ($p < 0.01$; **A**). Concordant to the result on RNA level, western blot analysis showed a decrease in protein expression ($p < 0.05$), confirming a successful downregulation (**B**).
